# MiR-526b-3p Attenuates Breast Cancer Stem Cell Properties and Chemoresistance by Targeting HIF-2α/Notch Signaling

**DOI:** 10.3389/fonc.2021.696269

**Published:** 2021-12-23

**Authors:** Jing-Hua Liu, Wen-Ting Li, Yue Yang, Yan-Bo Qi, Yu Cheng, Jia-Hui Wu

**Affiliations:** ^1^ Department of General Practice, School of Public Health, Qiqihar Medical University, Qiqihar, China; ^2^ Science Research Section, School of Public Health, Qiqihar Medical University, Qiqihar, China; ^3^ Teaching and Research Section, School of Public Health, Qiqihar Medical University, Qiqihar, China; ^4^ Qiqihar Medical University, Qiqihar, China; ^5^ Department of Nutrition and Food Hygiene, School of Public Health, Qiqihar Medical University, Qiqihar, China; ^6^ Department of Environmental and Occupational Health, School of Public Health, Qiqihar Medical University, Qiqihar, China

**Keywords:** breast cancer, hypoxia, CSCs, chemoresistance, miR-526b-3p, HIF-2α, Notch signaling

## Abstract

Chemoresistance is a severe clinical challenge in breast cancer. Hypoxia and cancer stem cells (CSCs) contribute to the paclitaxel (PTX) resistance, but the molecular mechanisms are still elusive. MicorRNAs (miRNA) have been considered a promising therapeutic strategy in various cancers. Here, we identified the crucial function of miR-526b-3p in regulating PTX resistance and CSC properties. Our data demonstrated that miR-526b-3p mimic repressed the cell viability of breast cancer cells. The counts of Edu-positive cells were reduced by miR-526b-3p in breast cancer cells. Meanwhile, the apoptosis of breast cancer cells was induced by miR-526b-3p. Tumorigenicity analysis in the nude mice confirmed that miR-526b-3p attenuated the breast cancer cell growth *in vivo.* Significantly, hypoxia could enhance IC_50_ value of PTX in breast cancer cells. IC_50_ value of PTX was induced in breast cancer mammospheres. The hypoxia-inducible factor 2α (HIF-2α) expression was enhanced, but miR-526b-3p expression was repressed under hypoxia in breast cancer cells. Also, breast cancer mammospheres presented high HIF-2α expression and low miR-526b-3p expression. The inhibition of miR-526b-3p enhanced the IC_50_ value of PTX in breast cancer cells. MiR-526b-3p inhibitor enhanced the colony formation counts of PTX-treated breast cancer cells. The treatment of miR-526b-3p mimic suppressed the sphere formation counts of breast cancer cells and inhibited ALDH1 and Nanog expression. MiR-526b-3p was able to target HIF-2α in the cells. The overexpression enhanced but miR-526b-3p reduced the IC_50_ value of PTX in breast cancer cells, in which the overexpression of HIF-2α could rescue the miR-526b-3p-inhibited IC_50_ value of PTX. Overexpression of HIF-2α reversed miR-526b-3p-regulated apoptosis, colony formation ability, and ALDH1 and Nanog expression in the cells. Interestingly, the overexpression of HIF-2α induced but miR-526b-3p repressed the expression of HIF-2α, Hey2, and Notch in PTX-treated breast cancer cells, while HIF-2α could reverse the effect of miR-526b-3p. In conclusion, miR-526b-3p attenuated breast cancer stem cell properties and chemoresistance by targeting HIF-2α/Notch signaling. MiR-526b-3p may be utilized in the relieving chemoresistance in breast cancer.

## Introduction

Breast cancer is one of the prevalent malignancies among females all over the world (1). The commonly applied clinical therapeutic approaches include the classic surgical procedures, endocrine therapy for estrogen receptor-positive phenotype, HER2-targeting therapy, and chemotherapy such as paclitaxel (PTX) (2). In clinical application, the efficacy of chemotherapeutic drugs largely varied among different patient groups, which is closely related with chemoresistance (2). Chemoresistance leads to impaired therapeutic response and even cancer recurrence, severely threatening the clinical prognosis and life quality of cancer patients (3). PTX is the most prevalent chemical drug for breast cancer treatment, which makes the chemoresistance against PTX a great challenge for breast cancer research (3). It is well accepted that chemoresistance mostly derives from the acquired resistance caused by gene mutations during long-term treatment (3). However, the concept of cancer stem cells (CSCs) has drawn great attention in research area of breast cancer chemoresistance (4).

Breast cancer stem cells (BCSCs) is a group of cancer cells with unique abilities of self-renewal, hence are thought to be responsible for initiation, progression, and the heterogeneity of breast cancer (5). The unique self-renewal ability of BCSCs confers a survival advantage under DNA damage caused by chemo- and radiotherapy and contributes to cancer recurrence (5). It has been revealed that the portion of BCSCs is notably elevated in those breast cancer cells resistant to chemo- and radiotherapy (6). Therefore, targeting BCSCs is a plausible way to overcome chemoresistance. Notch signaling is a critical regulator extensively reported in carcinogenesis of multiple cancers (7). It has been suggested that activation of Notch promoted critical downstream signaling such as Hey2 and c-Myc to participate in cancer stem cell function (8).

MicroRNAs (miRNAs) is a large group of endogenous short-length noncoding RNAs that could directly target and interact with mRNAs to manipulate gene expression (9). MiRNAs have been widely suggested as critical regulators during numerous biological processes including drug resistance of cancers (10–12). MiR-526b-3p is reported as a tumor suppressor in several cancers including metastatic colon cancer, glioma, and gastric cancer (13–15). Moreover, it was recently revealed to suppress cisplatin resistance of colorectal cancer cells by targeting KLF12 (16).

Hypoxia-inducible factor 2α (HIF-2α) is a member of hypoxia-inducible factor family, which is commonly elevated in cancer cells under hypoxia condition, capable of facilitating angiogenesis, metastasis, as well as promoting cancer cell stemness with a high specificity (17). It is reported that HIF-2α is involved in regulating self-renewal ability of BCSCs and participates in the chemoresistance to PTX (18).

In this research, we discovered that miR-526b-3p suppress breast cancer cell proliferation and resistance to PTX. The possible mechanisms involve direct interaction with HIF-2α and downstream Notch signaling transduction. This study provided a new approach to overcome PTX resistance in patients with breast cancer.

## Materials and Methods

### Cell Lines and Treatment

Human breast cancer cell lines were obtained from the Cell Bank of the Chinese Academy of Sciences (Shanghai, China). Cells were cultured with high glucose-DMEM (Hyclone, Logan, UT, USA) supplemented with 10% FBS (Hyclone) and 100 U/ml penicillin and 100 mg/L streptomycin (Solarbio, Beijing, China) and placed in a normal condition with 5% CO_2_. For culture of mammosphere, cells were suspended in serum-free DMEM/F12 medium (Hyclone) added with 2% B27 (Sigma, St. Louis, MO, USA), 20 ng/ml EGF (Sigma), and 20 ng/ml b-FGF (Sigma) in an ultra-low-attachment plate, and were incubated for 10 days. For hypoxia model, cells were placed in a 37°C incubator filled with 1% O_2_, 94% N_2_, and 5% CO_2_. The breast cancer stem cells were identified as CD44^+^CD24^−^ cells using fluorescence-activated cell sorting (FACS). MiR-526b-3p mimics and inhibitor and HIF-2α overexpressing vector (pcDNA-HIF-2α) were synthesized by GenePharma (Shanghai, China) and transfected to cell by using a Lipofectamine 2000 reagent (Invitrogen, Waltham, MA, USA).

### Cell Proliferation and Apoptosis

Cell proliferation of MCF-7 and MDA-MB-231 was determined by colony formation and EdU assay by using commercial kits (Beyotime, Haimen, China) following manufacturer’s protocols. Cell viability of MCF-7 and MDA-MB-231 was analyzed by CCK-8 assay by using commercial kits (Beyotime, China) following manufacturer’s protocols. In brief, cells were seeded in 96-well plates at a density of 5,000 cells per well, transfected with miR-526b-3p mimics, pcDNA-HIF-2α vector, or PTX at indicated doses. At indicated time points, 20-μl CCK-8 solution was added into each well and incubated for 2 h. The absorbance values at 450 nm were measured by a spectrophotometer (BioRad, Hercules, CA, USA). For EdU assay, proliferative cells were stained by EdU solution (50 µM) in the dark for 3 h, and nuclei were labeled by Hoechst 33342. The positive staining was captured by a fluorescence microscope (Leica, Wetzlar, Germany).

For colony formation experiment, cells were transfected with HIF-2α overexpressing vector or the empty vector and seeded in six-well plates at a density of 1,000 cells/well, cultured for 15 days till visible colonies are formed. The colonies were fixed in methanol, stained with crystal violet for 20 min, and subsequently photographed by a microscope (Leica).

The apoptotic cells were determined by an apoptotis-detecting kit obtained from Beyotime in accordance with manufacturer’s instruction. Briefly, cells were harvested after treatment, suspended in binding buffer, hatched with FITC-conjugated Annexin V (5 μl) and subsequent PI (5 μl) in the dark for 10 min. The samples were then analyzed in a flow cytometer (BD Biosciences, Franklin Lakes, NJ, USA).

### Mice Model

SCID/nude mice aged 6 weeks were purchased from Vital River Laboratory (China). MCF-7 cells were transfected with miR-526b-3p mimics/inhibitor or negative control and were subcutaneously injected into mice (2 × 10^6^ cells/mice), separately. The tumor size (width^2^ × length/2) and body weight were measured every other 2 days. At the end time, mice were killed and the tumors were weighted and collected for further experiments. The animal study was reviewed and approved by Qiqihar Medical University.

### Quantitative Real-Time PCR

MCF-7 and MDA-MB-231 cells were lysed with Trizol reagent (Sigma, USA) to extract total RNA. The total RNA was then subjected to reverse transcription by using PrimeScript RT reagent kit (Takara, Kusatsu, Japan), and the subsequent quantification by SYBR Green labeling (Takara). The relative expression of mRNAs and miR-526b-3p were normalized to GAPDH and U6, respectively, and calculated with a 2^−△△Ct^ method. The primers were listed as follows:

miR-526b-3p: forward: 5′-GCGCTCTTGAGGGAAGCACT-3′, reverse: 5′-TACGTTCCATAGTCTACCA-3′;
HIF-2α: forward: ACCATGCCCCAGATTCAGG, reverse: AGTGCTTCCATCGGAAGGACT;
ALDH1: forward: 5′-CACCTCGCTGGAGTACGGA-3′, reverse: 5′-CCATTCACATAGTGGCCCAAG-3′;
Nanog: forward: 5′-TTTGTGGGCCTGAAGAAAACT-3′, reverse: 5′-AGGGCTGTCCTGAATAAGCAG-3′;
GAPDH: forward: 5′-GGTCTCCTCTGACTTCAACA-3′, reverse: 5′-GCCAAATTCGTTGTCATAC-3′.

### Western Blotting Assay

Cells were collected after indicated treatment and lysed in RIPA buffer added with proteinase inhibitor cocktail to obtain total proteins. A total of 30 μg protein was divided in SDS-PAGE gels, blotted to NC membranes, and interacted with specific primary antibodies including anti-ALDH1 (Abcam, Cambridge, MA, USA, 1:1,000), anti-Nanog (Abcam, USA, 1:1,000), anti-HIF-2α (Abcam, USA, 1:1,000), anti-Notch (Abcam, USA, 1:1,000), anti-Hey2 (Abcam, USA, 1:1,000), and anti-GAPDH (Abcam, USA, 1:1,000). The protein bands were visualized by incubating with HRP-conjugated secondary antibodies and an ECL solution (Millipore, USA). All antibodies were ordered from Abcam and used following manufacturer’s description.

### Luciferase Reporter Gene Assay

The binding site of miR-526b-3p on the mRNA region of HIF-2α was predicted *via* the TargetScan website (http://www.targetscan.org/vert_71/). The wild-type (WT) and mutated (MUT) sequences of 3′UTR of HIF-2α were cloned into pGL3-basic plasmid (Promega, Madison, WI, USA). The Renilla was adopted as reference. WT or MUT plasmids were cotransfected with pRLTK and miR-526b-3p into MCF-7 cells and incubated for 48 h. Cells were then collected and lysed, and the luciferase activity was measured by a dual luciferase viability detection kit (Promega).

### Statistical Analysis

All data were shown as means ± SD and analyzed by using SPSS software (version 21). Student’s *t*-test was used for comparison between two groups, and the one-way ANOVA was used for multiple comparisons. Differences with *p* < 0.05 were considered statistically significant.

## Results

### MiR-526b-3p Suppresses Proliferation and Enhances Apoptosis of Breast Cells

**Figure 1:**
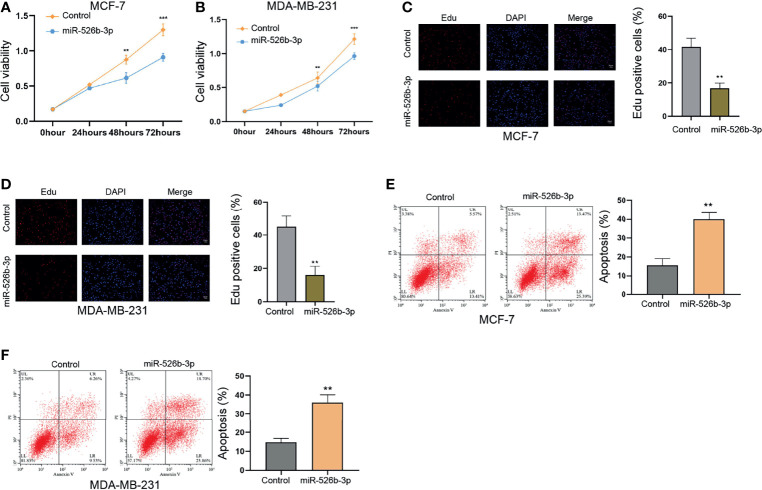
MiR-526b-3p suppresses proliferation and enhances apoptosis of breast cells *in vitro.* **(A–F)** The MCF-7 and MDA-MB-231 cells were transfected with miR-526b-3p mimic. **(A, B)** Viability of MCF-7 and MDA-MB-231 cells was detected by CCK-8 analysis. **(C, D)** Proliferation of MCF-7 and MDA-MB-231 cells was analyzed by Edu analysis. **(E, F)** Apoptosis of MCF-7 and MDA-MB-231 cells was determined by flow cytometry analysis. Data are mean ± SD: ^**^ *p* < 0.01, ^***^ *p* < 0.001.

We observed that the expression of miR-526b-3p was reduced in the indicated breast cancer cells (**Supplementary Figure S1A**). The association of miR-526b-3p with breast cancer has not been identified; we thereby determined the effect of miR-526b-3p on breast cancer cells. Significantly, the enhancement of miR-526b-3p by miR-526b-3p mimic in MCF-7 and MDA-MB-231 cells repressed the cell viability (**Figures 1A, B** **)**. The counts of Edu-positive cells were reduced by miR-526b-3p in MCF-7 and MDA-MB-231 cells (**Figures 1C, D** **)**. Meanwhile, the apoptosis of MCF-7 and MDA-MB-231 cells was induced by miR-526b-3p as well (**Figures 1E, F** **)**.

**Figure 2:**
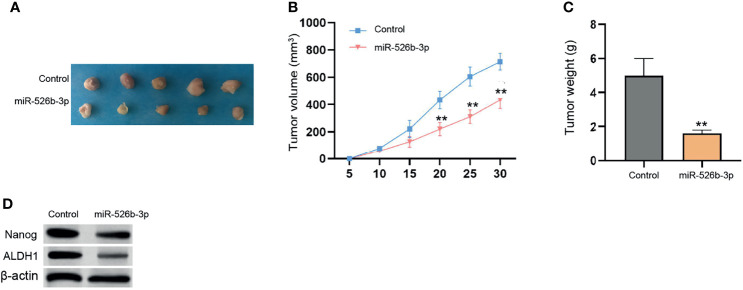
MiR-526b-3p attenuates breast cancer cell growth *in vivo*. **(A**–**D)** The nude mice (*n* = 5) were injected with MCF-7 cells transfected with control mimic or miR-526b-3p mimic. The tumorigenicity *in vivo* was observed. The representative tumor tissues **(A)**, tumor volume **(B)**, and weight **(C)** were presented. Data are mean ± SD: ^**^ *p* < 0.01.

Next, the tumorigenicity analysis in the nude mice (*n* = 5) confirmed that the treatment with miR-526b-3p could attenuate the MCF-7 cell growth and tumor volume and weight *in vivo* (**Figure 2**). In addition, the protein levels of ALDH1 and Nanog in MCF-7 and MDA-MB-231 cells were decreased by miR-526b-3p in the mice (**Figure 2**). Meanwhile, the inhibitor of miR-526b-3p presented a reversed effect in the model (**Supplementary Figure S2**).

### Hypoxia Enhances PTX Resistance and HIF-2α Expression and Reduces miR-526b-3p Expression in Breast Cancer Cells

**Figure 3:**
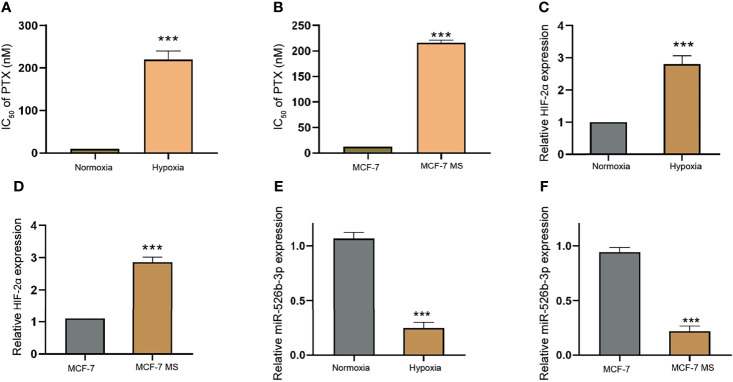
Hypoxia enhances PTX resistance and HIF-2α expression and reduces miR-526b-3p expression in breast cancer cells. **(A)** IC_50_ value of PTX was compared in MCF-7 cells under hypoxia or normoxia condition. **(B)** IC_50_ value of PTX was compared in MCF-7 cells and MCF-7 mammospheres (MCF-7 MS). **(C)** The expression of HIF-2α was evaluated by qPCR in MCF-7 cells under hypoxia or normoxia condition. **(D)** The expression of HIF-2α was assessed by qPCR in MCF-7 cells and MCF-7 MS. **(E)** The expression of miR-526b-3p was evaluated by qPCR in MCF-7 cells under hypoxia or normoxia condition. **(F)** The expression of miR-526b-3p was assessed by qPCR in MCF-7 cells and MCF-7 MS. Data are mean ± SD: ^***^ *p* < 0.001.

Next, we assessed the correlation of miR-526b-3p with hypoxia and PTX resistance in breast cancer cells. We firstly evaluated the effect of hypoxia on PTX resistance by detecting cell viability of MCF-7 treated with different concentrations of PTX under hypoxia or normoxia condition. Significantly, hypoxia could enhance IC_50_ value of PTX in MCF-7 and MDA-MB-231 cells (**Figure 3A** and **Supplementary Figure S3A**). Given that hypoxia affects the CSC properties of breast cancer, we further determined the IC_50_ value of PTX in MCF-7 mammospheres (MCF-7 MS) and MDA-MB-231 mammospheres (MDA-MB-231-MS). We found that the IC_50_ value of PTX was induced in MCF-7 MS and MDA-MB-231-MS relative to MCF-7 and MDA-MB-231 cells (**Figure 3B** and **Supplementary Figure S3B**). Then, we validated that the expression of HIF-2α was enhanced under hypoxia in MCF-7 and MDA-MB-231 cells (**Figure 3C** and **Supplementary Figure S3C**). Similarly, MCF-7 MS and MDA-MB-231-MS presented higher expression of HIF-2α than MCF-7 and MDA-MB-231 cells (**Figure 3D** and **Supplementary Figure S3D**). Importantly, the expression of miR-526b-3p was repressed in hypoxia MCF-7 MDA-MB-231cells and MCF-7 MS (**Figures 3E, F** and **Supplementary Figures S3E, F**).

### MiR-526b-3p Represses PTX Resistance of Breast Cancer Cells

**Figure 4:**
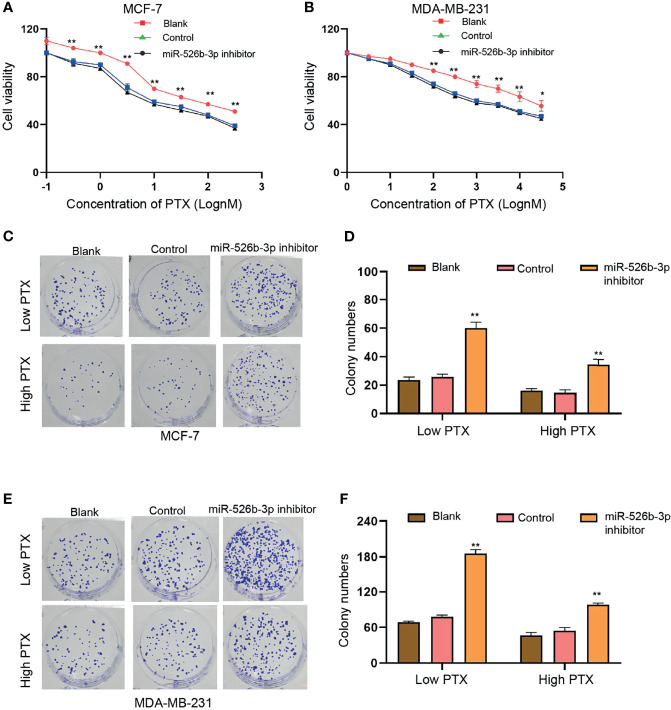
MiR-526b-3p represses PTX resistance of breast cancer cells. **(A**–**F)** MCF-7 and MDA-MB-231 cells were treated with different concentration of PTX and were co-treated with miR-526b-3p inhibitor. **(A, B)** Viability of MCF-7 and MDA-MB-231 cells was detected by CCK-8 analysis. **(C–F)** MCF-7 and MDA-MB-231 cells were treated with low dose (3 nM) or high dose (100 nM) of PTX and were cotransfected with miR-526b-3p inhibitor. Proliferation of MCF-7 and MDA-MB-231 cells was detected by colony formation assays. Data are mean ± SD: ^*^ *p* < 0.05, ^**^ *p* < 0.01.

Next, we further explored the effect of miR-526b-3p on PTX resistance in breast cancer cells. We observed that the suppression of miR-526b-3p by miR-526b-3p inhibitor significantly enhanced the IC_50_ value of PTX in MCF-7 and MDA-MB-231 cells (**Figures 4A, B** **)**. Meanwhile, the treatment of high concentration of PTX (100 nM) repressed the colony formation counts compared with the treatment of low concentration of PTX (3 nM) in MCF-7 and MDA-MB-231 cells (**Figures 4C–E**). Moreover, the treatment of miR-526b-3p inhibitor enhanced the colony formation counts of MCF-7 and MDA-MB-231 cells in the system (**Figures 4C–F**).

### MiR-526b-3p Suppresses CSC properties of Breast Cancer Cells *In Vitro*

**Figure 5:**
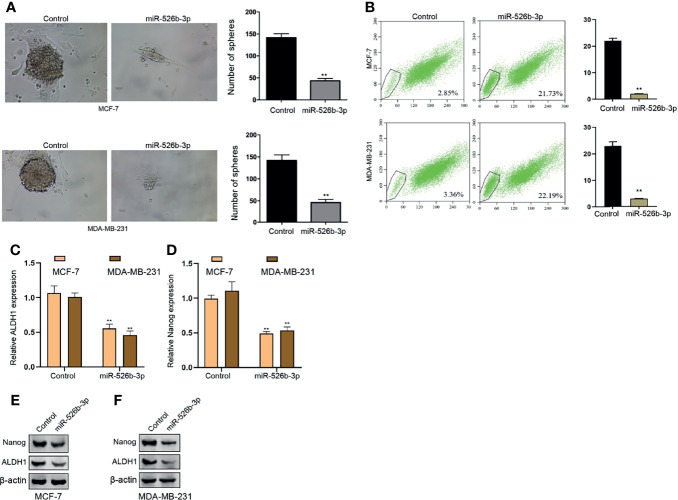
MiR-526b-3p suppresses CSCs properties of breast cancer cells *in vitro*. **(A**–**F)** The MCF-7 and MDA-MB-231 cells were transfected with miR-526b-3p mimic. **(A)** Stemness of MCF-7 and MDA-MB-231 cells was analyzed by sphere formation assays. **(B)** The SP ratio of MCF-7 and MDA-MB-231 cells was measured by flow cytometry analysis of Hoechst 33342 staining. **(C, D)** The mRNA expression of ALDH1 and Nanog was measured by qPCR. **(E, F)** The protein expression of ALDH1 and Nanog was detected by Western blot. Data are mean ± SD: ^**^ *p* < 0.01.

Then, we tried to determine the function of miR-526b-3p in the modulation of CSCs properties of breast cancer cells. We observed that the expression of miR-526b-3p was reduced in breast cancer stem cells (**Supplementary Figure S1B**). Our data demonstrated that the treatment of miR-526b-3p mimic suppressed the sphere formation counts and SP ratio of MCF-7 and MDA-MB-231 cells (**Figures 5A, B** **)**. Meanwhile, the enhancement of miR-526b-3p repressed mRNA expression of ALDH1 and Nanog in MCF-7 and MDA-MB-231 cells (**Figures 5C, D** **)**. Meanwhile, the protein levels of ALDH1 and Nanog in MCF-7 and MDA-MB-231 cells were decreased by miR-526b-3p as well (**Figures 5E, F** **)**.

### MiR-526b-3p Can Target HIF-2α in Breast Cancer Cells

**Figure 6:**
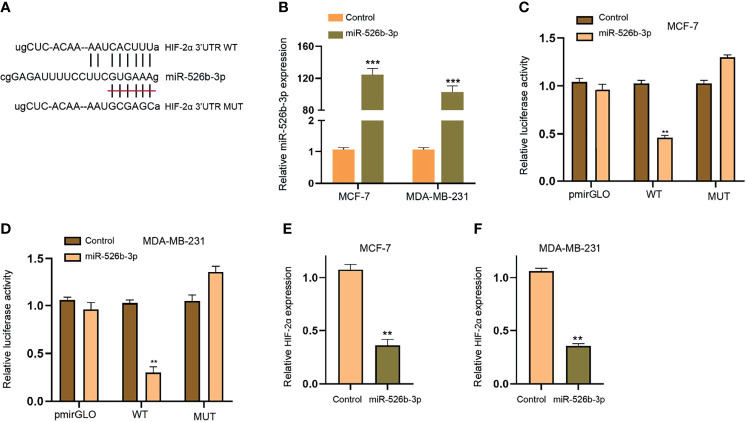
MiR-526b-3p can target HIF-2α in breast cancer cells. **(A)** The binding site (WT) and mutant binding site (MUT) of HIF-2α 3′UTR with miR-526b-3p was shown. **(B**–**F)** The MCF-7 and MDA-MB-231 cells were transfected with miR-526b-3p mimic. **(B)** The expression of miR-526b-3p was validated by qPCR. **(C, D)** The luciferase activity of HIF-2α 3′UTR was detected by luciferase reporter gene assays. **(E, F)** The expression of HIF-2α was assessed by qPCR. Data are mean ± SD: ^**^ *p* < 0.01, ***p < 0.001.

Next, we focused on the correlation of miR-526b-3p with HIF-2α in breast cancer cells. We found the binding site between miR-526b-3p and HIF-2α 3′UTR and constructed the miR-526b-3p-binding site mutant HIF-2α 3′UTR (**Figure 6A**). The enhancement of miR-526b-3p by miR-526b-3p mimic was confirmed in MCF-7 and MDA-MB-231 cells (**Figure 6B**). Meanwhile, the miR-526b-3p mimic repressed the luciferase activity of HIF-2α but failed to change the luciferase activity of HIF-2α mutant (**Figures 6C, D** **)**. Consistently, the mRNA expression of HIF-2α was inhibited by miR-526b-3p in MCF-7 and MDA-MB-231 cells (**Figures 6E, F** **)**.

### MiR-526b-3p Attenuates PTX Resistance of Breast Cancer Cells by Targeting HIF-2α

**Figure 7:**
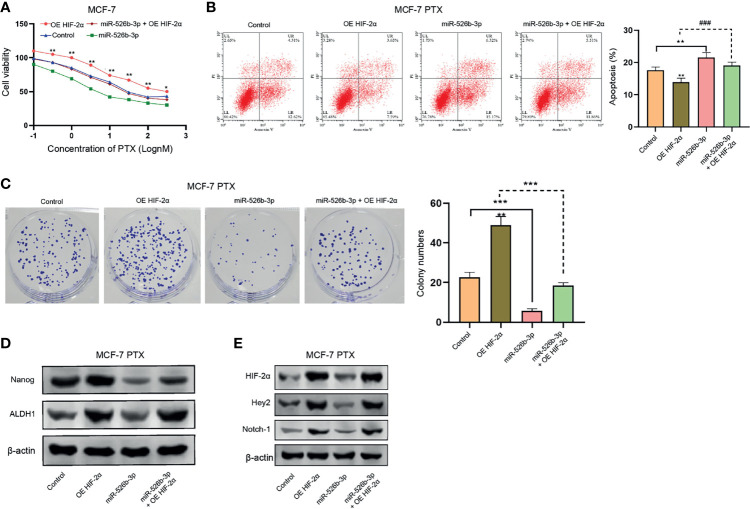
MiR-526b-3p attenuates PTX resistance of breast cancer cells by targeting HIF-2α**. (A)** MCF-7 cells were treated with different concentrations of PTX and were cotreated with miR-526b-3p mimic or HIF-2α overexpression plasmid. Viability of MCF-7 cells was detected by MTT analysis. **(B**–**E)** MCF-7 cells were treated with PTX (3 nM) and cotreated with miR-526b-3p mimic or HIF-2α overexpression plasmid. **(B)** Apoptosis of MCF-7 cells was determined by flow cytometry analysis. **(C)** Proliferation of MCF-7 cells was detected by colony formation assays. **(D)** The protein expression of ALDH1 and Nanog was tested by Western blot. **(E)** The protein expression of HIF-2α, Hey2, and Notch-1 was assessed by Western blot. Data are mean ± SD: ^*^ *p* < 0.05, ^**^ *p* < 0.01, ***p < 0.001; ^###^ *p* < 0.001.

We then investigated the function of miR-526b-3p/HIF-2α axis in regulating PTX resistance of breast cancer cells. Our data showed that the overexpression of HIF-2α enhanced but miR-526b-3p reduced the IC_50_ value of PTX in MCF-7 cells, in which the overexpression of HIF-2α could rescue the miR-526b-3p-inhibited IC_50_ value of PTX (**Figure 7A**). Moreover, we confirmed that miR-526b-3p repressed and HIF-2α overexpression enhanced apoptosis of PTX-treated MCF-7 cells, and HIF-2α overexpression could block the enchantment of apoptosis induced by miR-526b-3p (**Figure 7B**). Meanwhile, the colony formation counts of PTX-treated MCF-7 cells were inhibited by miR-526b-3p and increased by HIF-2α, while HIF-2α could reverse the effect of miR-526b-3p (**Figure 7C**). The protein levels of ALDH1 and Nanog were upregulated by HIF-2α but downregulated by miR-526b-3p in PTX-treated MCF-7 cells, in which the overexpression of HIF-2α rescued the expression suppressed by miR-526b-3p (**Figure 7D**). Given the reported correlation of HIF-2α with Notch signaling, we were concerned about whether miR-526b-3p could regulate Notch signaling in breast cancer cells. Our data confirmed that the overexpression of HIF-2α induced but miR-526b-3p repressed the expression of HIF-2α, Hey2, and Notch-1 in PTX-treated MCF-7 cells, while HIF-2α could reverse the effect of miR-526b-3p (**Figure 7E**).

## Discussion

Breast cancer is a common female malignancy and PTX resistance is a severe clinical problem in the treatment of breast cancer. Hypoxia is closely correlated with CSCs and contributes to the PTX resistance. However, the molecular mechanisms are still elusive. In this research, we identified the crucial function of miR-526b-3p in regulating PTX resistance and CSC properties.

Previous studies have presented the obvious association of hypoxia and CSCs with chemoresistance in breast cancer. HIF-2α contributes to CSC progression and chemoresistance by activating Notch signaling in breast cancer (19). STAT3 is involved in the regulation of hypoxia-stimulated chemoresistance in breast cancer (20). Hypoxia contributes to drug resistance in breast cancers (21). Hypoxia-associated anticancer drug resistance is correlated with senescence and HIF1 in breast cancer (22). Moreover, it has been found that miR-526b-3p serves as a cancer inhibitor by regulating HIF-1α in colon cancer (13). MiR-526b-3p is a prognostic biomarker and modulates the progression of glioma by targeting WEE1 (23). YY1 contributes to colorectal cancer development by the miR-526b-3p/E2F1 signaling (24). In this research, we showed that miR-526b-3p mimic repressed the cell proliferation of breast cancer cells. Meanwhile, the apoptosis of breast cancer cells was induced by miR-526b-3p. Tumorigenicity analysis in the nude mice confirmed that miR-526b-3p attenuated the breast cancer cell growth *in vivo.* Hypoxia could enhance IC_50_ value of PTX in breast cancer cells. IC_50_ value of PTX was induced in breast cancer mammospheres. The HIF-2α expression was enhanced, but miR-526b-3p expression was repressed under hypoxia in breast cancer cells. The inhibition of miR-526b-3p enhanced the IC_50_ value of PTX in breast cancer cells. MiR-526b-3p inhibitor enhanced the colony formation counts of PTX-treated breast cancer cells. The treatment of miR-526b-3p mimic suppressed the CSC properties of breast cancer cells. These data suggest that miR-526b-3p is an innovative regulator of CSC properties and PTX resistance of breast cancer cells. The clinical correlation of miR-526b-3p with breast cancer is still unclear. Meanwhile, the application of miR-526b-3p for the attenuation of chemoresistance of breast cancer in the clinic needs to resolve the problem of miR-526b-3p delivery.

HIF-2α and Notch signaling play critical roles in the regulation of CSC properties and PTX resistance in breast cancer. Hypoxia enhances breast cancer by ALKBH5-mediated and HIF-dependent Nanog mRNA m⁶A-demethylation (25). High aldehyde dehydrogenase activity contributes to the stemness of breast cancer cells by inducing HIF-2α (26). BMP-4 promotes CSC properties and epithelial mesenchymal transition by Notch signaling in breast cancer (27). MiR-34a represses the stemness of breast cancer cells by downregulating Notch signaling (28). HIF-2α modulates CD44 to enhance activation of CSCs by PI3K/AKT/mTOR signaling in breast cancer (29). Here, we presented that miR-526b-3p was able to target HIF-2α in the breast cancer cells. MiR-526b-3p attenuated PTX resistance and CSC properties of breast cancer cells by targeting HIF-2α. The overexpression of HIF-2α induced but miR-526b-3p repressed the expression of HIF-2α, Hey2, and Notch in PTX-treated breast cancer cells, while HIF-2α could reverse the effect of miR-526b-3p. These data imply that miR-526b-3p may exert its function in breast cancer by regulating HIF-2α/Notch signaling. More experimental evidence is required to prove the association of miR-526b-3p with Notch signaling in the modulation of PTX resistance and CSC properties in breast cancer.

In conclusion, miR-526b-3p attenuated breast cancer stem cell properties and chemoresistance by targeting HIF-2α/Notch signaling. MiR-526b-3p may be utilized in relieving chemoresistance in breast cancer.

## Data Availability Statement

The original contributions presented in the study are included in the article/**Supplementary Material**. Further inquiries can be directed to the corresponding author.

## Ethics Statement

The animal study was reviewed and approved by Qiqihar Medical University.

## Author Contributions

J-HL and W-TL designed the experiments. YY and YC performed the experiments and analyzed the data. Y-BQ and J-HW performed analysis and wrote the paper.

## Funding

This study was supported by the scientific research project of Qiqihar Science and Technology Bureau, SFGG-201907.

## Conflict of Interest

The authors declare that the research was conducted in the absence of any commercial or financial relationships that could be construed as a potential conflict of interest.

## Publisher’s Note

All claims expressed in this article are solely those of the authors and do not necessarily represent those of their affiliated organizations, or those of the publisher, the editors and the reviewers. Any product that may be evaluated in this article, or claim that may be made by its manufacturer, is not guaranteed or endorsed by the publisher.

## Supplementary Material

Supplementary Figure 1 The expression of miR-526b-3p in breast cancer cells. **(A, B)** The expression of miR-526b-3p was assessed by qPCR in the indicated cells. Data are mean ± SD: ****P* < 0.001.

Supplementary Figure 2 MiR-526b-3p inhibitor promotes breast cancer cell growth *in vivo*. **(A–D)** The nude mice (n=5) were injected with MCF-7 cells transfected with control inhibitor or miR-526b-3p inhibitor. The tumorigenicity *in vivo* was observe. The representative tumor tissues **(A)**, tumor volume **(B)**, and weight **(C)** were presented. Data are mean ± SD: ***P* < 0.01.
