# Supplementary figures and images for: MiR-526b-3p Attenuates Breast Cancer Stem Cell Properties and Chemoresistance by Targeting HIF-2α/Notch Signaling
Source: Front Oncol. 2021 Dec 23;11:696269. doi: 10.3389/fonc.2021.696269 (PMC8733566; doi:10.3389/fonc.2021.696269)

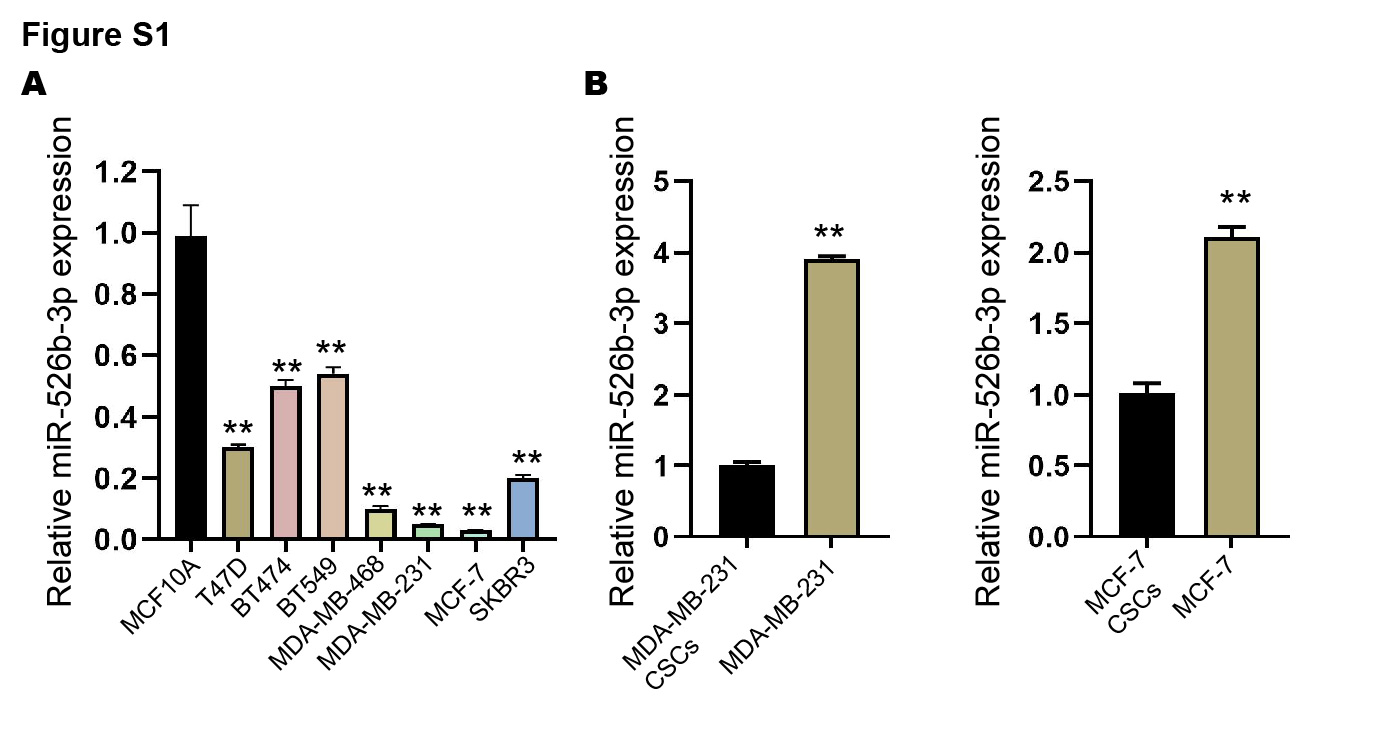

Supplement: Supplementary Figure 1 — The expression of miR-526b-3p in breast cancer cells. (A, B) The expression of miR-526b-3p was assessed by qPCR in the indicated cells. Data are mean ± SD: ***P < 0.001. [file Image_1.jpeg]

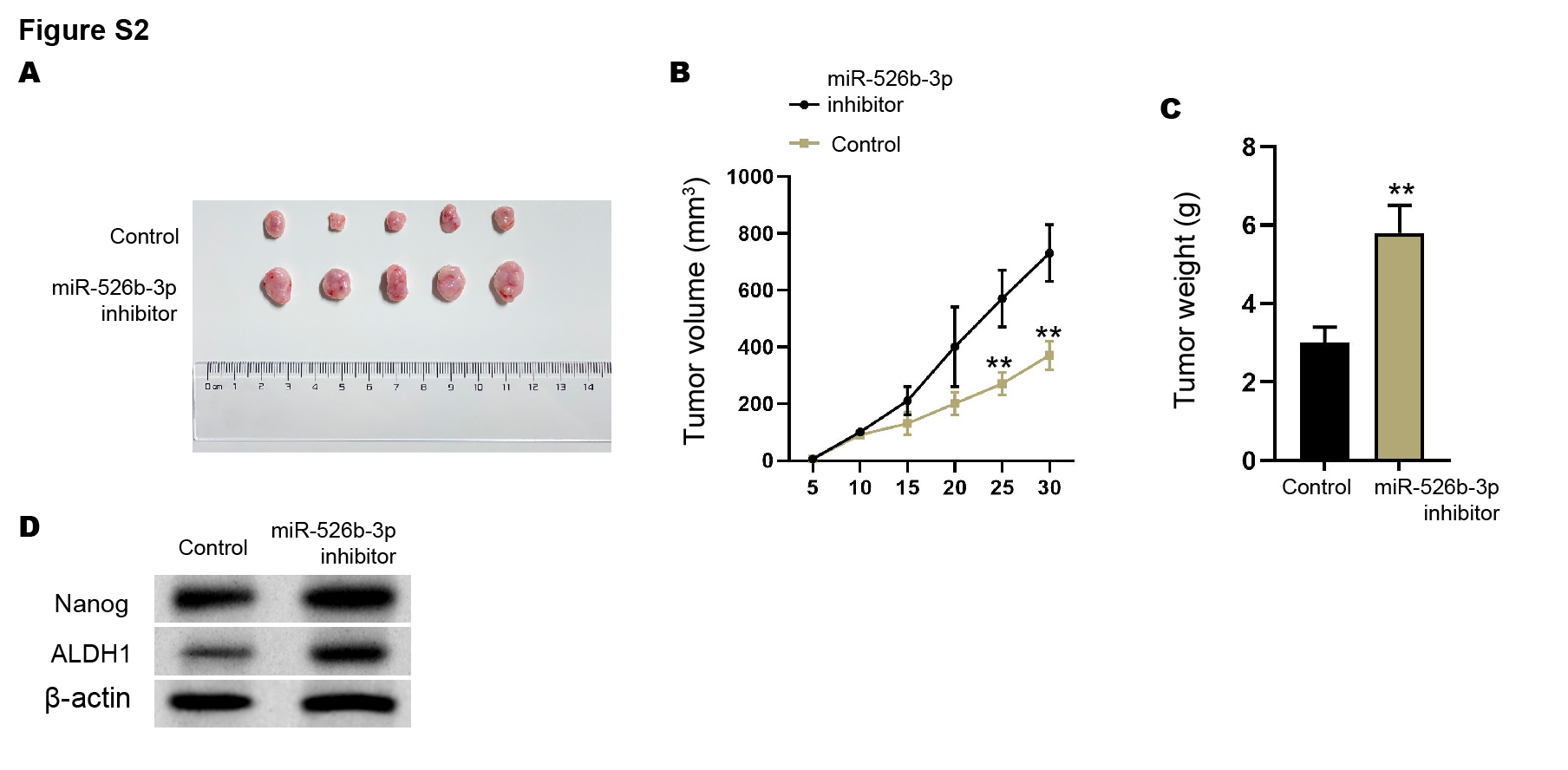

Supplement: Supplementary Figure 2 — MiR-526b-3p inhibitor promotes breast cancer cell growth in vivo. (A–D) The nude mice (n=5) were injected with MCF-7 cells transfected with control inhibitor or miR-526b-3p inhibitor. The tumorigenicity in vivo was observe. The representative tumor tissues (A), tumor volume (B), and weight (C) were presented. Data are mean ± SD: **P < 0.01. [file Image_2.jpeg]

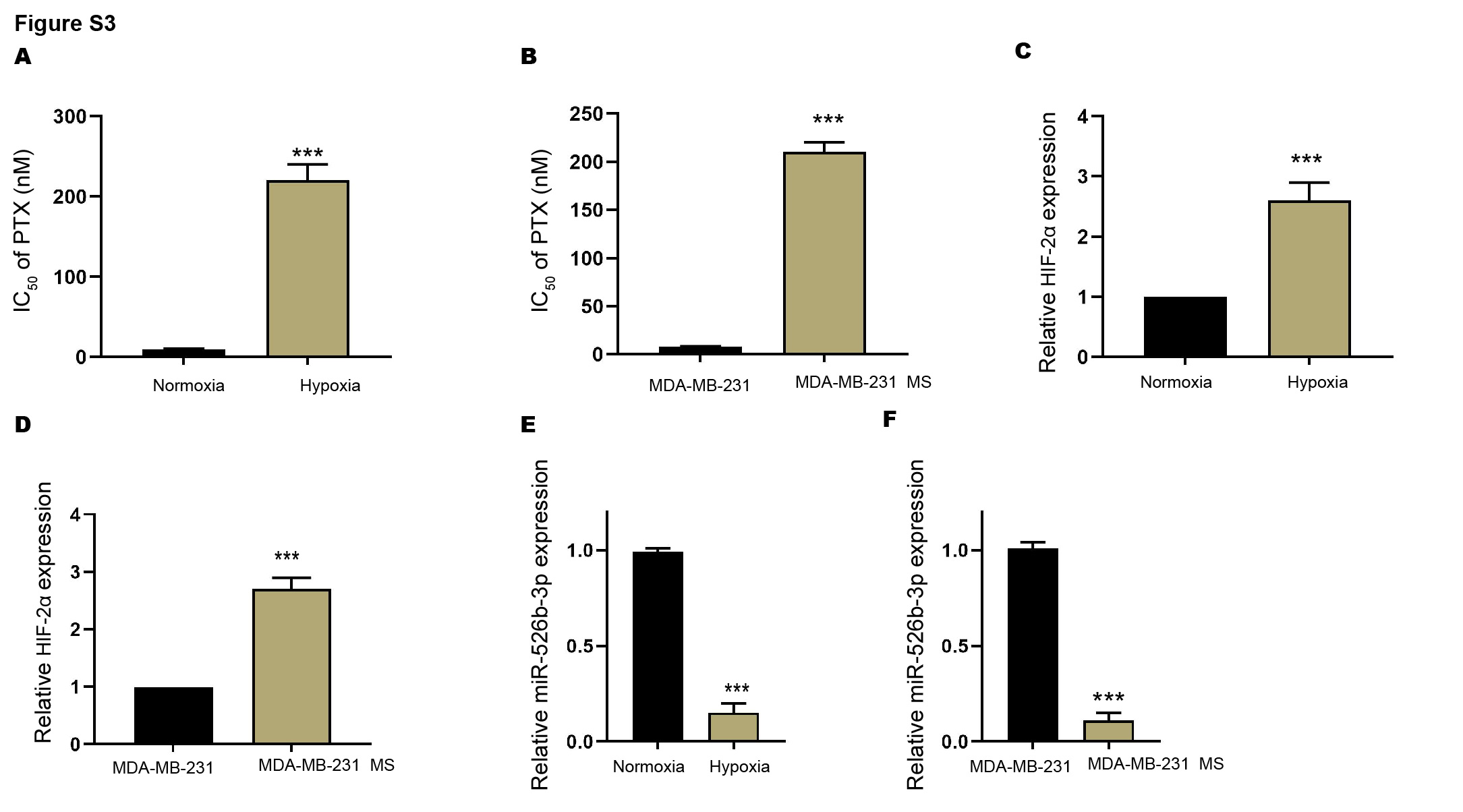

Supplement: Supplementary file 3 [file Image_3.jpeg]
